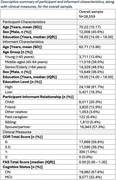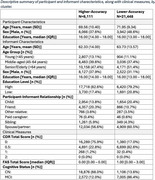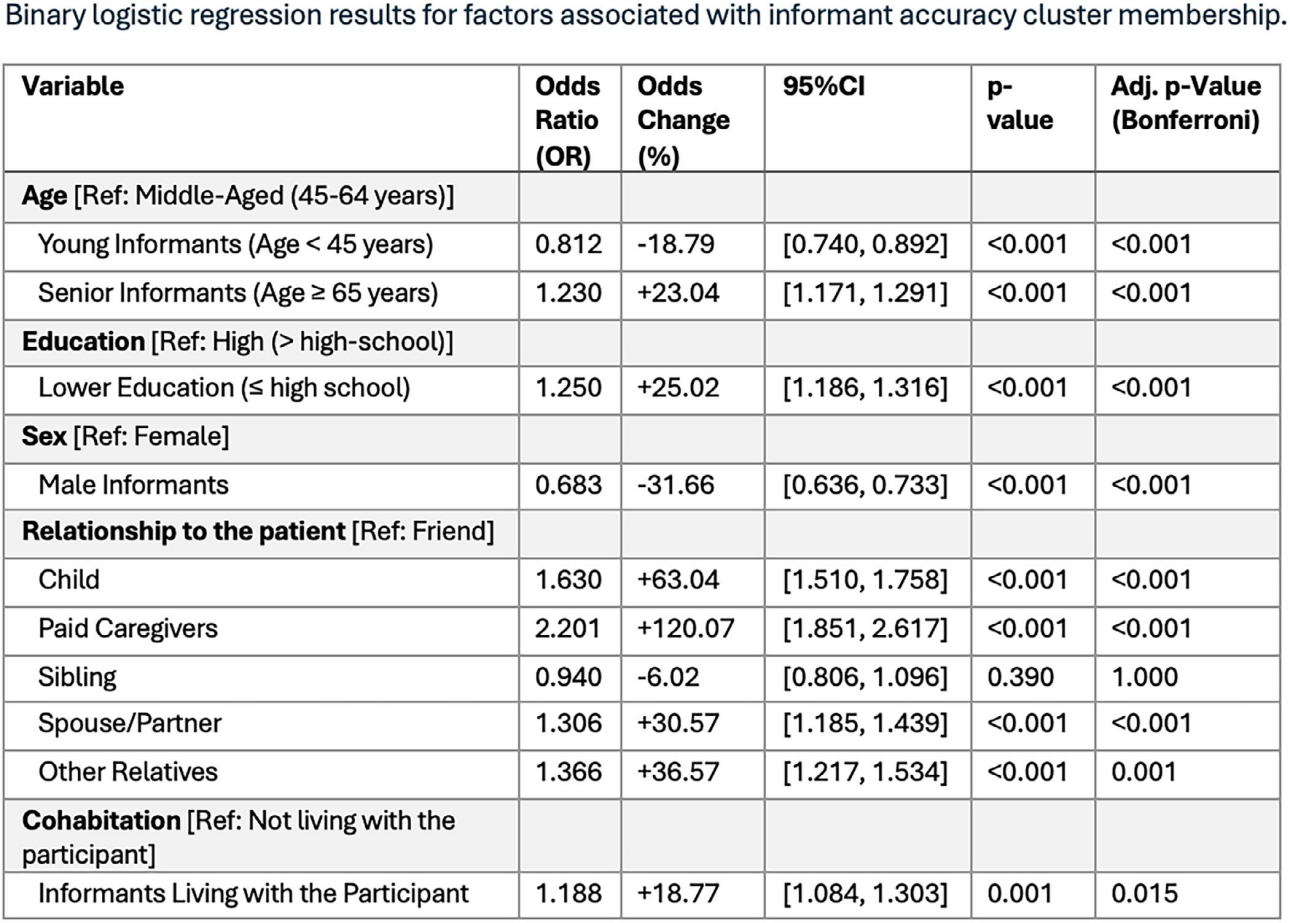# Leveraging informant perspectives to evaluate cognitive decline: Key characteristics of ideal informants

**DOI:** 10.1002/alz70857_101112

**Published:** 2025-12-25

**Authors:** Federica Cacciamani, Sophie Tezenas du Montcel

**Affiliations:** ^1^ Qairnel, Paris, France; ^2^ Paris Brain Institute, Sorbonne, CNRS, Inserm, Paris, France; ^3^ APHP, Paris, France

## Abstract

**Background:**

Informants are a critical source of information in clinical and research settings, especially when patients, as in Alzheimer's disease (AD), have reduced self‐awareness. However, little is known about which informants are best able to detect early changes in patients. This study examines the extent to which informant‐reported assessments align with clinician‐assessed cognitive status (mild cognitive impairment, MCI vs. cognitively normal, CN) and investigates how informant characteristics influence their accuracy.

**Method:**

We used baseline data from the NACC database (*N* = 28,559). Cognitive status (CN or MCI) was determined through clinical assessments. Informants completed the Functional Activities Scale (FAS) to evaluate patients’ abilities in daily activities. Residuals from a logistic regression model, assessing association between FAS and cognitive status, quantified informant accuracy. These residuals were used as input for k‐means clustering to classify informants into accuracy‐based clusters. A logistic regression was then conducted to compare informants’ characteristics (age, sex, education, relationship, cohabitation) in the clusters, using Bonferroni correction.

**Result:**

FAS was significantly associated with cognitive status (OR=1.37, *p* <0.001). Two clusters were identified: Higher‐Accuracy (28.4%) and Lower‐Accuracy (71.6%). Children (OR=1.63, +63.0%, *p* <0.001), other relatives (OR=1.37, +36.6%, *p* = 0.001), and spouses/partners (OR=1.31, +31.6%, *p* <0.001) were significantly more likely to belong to the Higher‐Accuracy cluster compared to friends. Paid caregivers (OR=2.20, +120.1%, *p* <0.001) also showed high odds of accuracy, though they represented a small subgroup. Siblings showed no significant difference (OR=0.94, *p* = 1.000). Men were 31.6% less likely to belong to the Higher‐Accuracy cluster compared to women (OR=0.68, *p* <0.001). Younger informants (<45 years) were less likely to belong to the Higher‐Accuracy cluster (OR=0.81, ‐18.9%, *p* <0.001), while senior/elderly informants (≥65 years) were more likely (OR=1.23, +23.0%, *p* <0.001). Informants with lower education (≤high school) had 25% higher odds of being in the Higher‐Accuracy cluster vs. those with higher education (OR=1.25, *p* <0.001). Informants living with the participant were 18.8% more likely to belong to the Higher‐Accuracy cluster (OR=1.19, *p* = 0.015).

**Conclusion:**

Informant characteristics significantly influence the alignment of their reports with clinician‐assessed cognitive status. These findings highlight the importance of selecting informants with specific profiles (e.g., patient's children, females), to enhance early detection and intervention in AD research and clinical practice.